# Comparison of *International Classification of Diseases and Related Health Problems, Tenth Revision* Codes With Electronic Medical Records Among Patients With Symptoms of Coronavirus Disease 2019

**DOI:** 10.1001/jamanetworkopen.2020.17703

**Published:** 2020-08-14

**Authors:** Brendan T. Crabb, Ann Lyons, Margaret Bale, Valerie Martin, Ben Berger, Sara Mann, William B. West, Alyssa Brown, Jordan B. Peacock, Daniel T. Leung, Rashmee U. Shah

**Affiliations:** 1 Department of Internal Medicine, University of Utah School of Medicine, Salt Lake City; 2 Data Science Services, University of Utah Health Sciences Center, Salt Lake City; 3 Division of Infectious Diseases, University of Utah School of Medicine, Salt Lake City; 4 Division of Cardiovascular Medicine, University of Utah School of Medicine, Salt Lake City

## Abstract

**Importance:**

*International Statistical Classification of Diseases and Related Health Problems, Tenth Revision *(*ICD-10*) codes are used to characterize coronavirus disease 2019 (COVID-19)–related symptoms. Their accuracy is unknown, which could affect downstream analyses.

**Objective:**

To compare the performance of fever-, cough-, and dyspnea-specific *ICD-10* codes with medical record review among patients tested for COVID-19.

**Design, Setting, and Participants:**

This cohort study included patients who underwent quantitative reverse transcriptase–polymerase chain reaction testing for severe acute respiratory syndrome coronavirus 2 at University of Utah Health from March 10 to April 6, 2020. Data analysis was performed in April 2020.

**Main Outcomes and Measures:**

The sensitivity, specificity, positive predictive value (PPV), and negative predictive value (NPV) of *ICD-10* codes for fever (R50*), cough (R05*), and dyspnea (R06.0*) were compared with manual medical record review. Performance was calculated overall and stratified by COVID-19 test result, sex, age group (<50, 50-64, and >64 years), and inpatient status. Bootstrapping was used to generate 95% CIs, and Pearson χ^2^ tests were used to compare different subgroups.

**Results:**

Among 2201 patients tested for COVD-19, the mean (SD) age was 42 (17) years; 1201 (55%) were female, 1569 (71%) were White, and 282 (13%) were Hispanic or Latino. The prevalence of fever was 66% (1444 patients), that of cough was 88% (1930 patients), and that of dyspnea was 64% (1399 patients). For fever, the sensitivity of *ICD-10* codes was 0.26 (95% CI, 0.24-0.29), specificity was 0.98 (95% CI, 0.96-0.99), PPV was 0.96 (95% CI, 0.93-0.97), and NPV was 0.41 (95% CI, 0.39-0.43). For cough, the sensitivity of *ICD-10* codes was 0.44 (95% CI, 0.42-0.46), specificity was 0.88 (95% CI, 0.84-0.92), PPV was 0.96 (95% CI, 0.95-0.97), and NPV was 0.18 (95% CI, 0.16-0.20). For dyspnea, the sensitivity of *ICD-10* codes was 0.24 (95% CI, 0.22-0.26), specificity was 0.97 (95% CI, 0.96-0.98), PPV was 0.93 (95% CI, 0.90-0.96), and NPV was 0.42 (95% CI, 0.40-0.44). *ICD-10* code performance was better for inpatients than for outpatients for fever (χ^2^ = 41.30; *P* < .001) and dyspnea (χ^2^ = 14.25; *P* = .003) but not for cough (χ^2^ = 5.13; *P* = .16).

**Conclusions and Relevance:**

These findings suggest that *ICD-10* codes lack sensitivity and have poor NPV for symptoms associated with COVID-19. This inaccuracy has implications for any downstream data model, scientific discovery, or surveillance that relies on these codes.

## Key Points

**Question:**

Do *International Statistical Classification of Diseases and Related Health Problems, Tenth Revision *(*ICD-10*) codes accurately capture presenting symptoms of fever, cough, and dyspnea among patients being tested for coronavirus disease 2019 (COVID-19)?

**Findings:**

In this cohort study, an electronic medical record review of 2201 patients tested for COVID-19 between March 10 and April 6, 2020, found that *ICD-10* codes had poor sensitivity and negative predictive value for capturing fever, cough, and dyspnea.

**Meaning:**

These findings suggest that symptom-specific *ICD-10* codes do not accurately capture COVID-19–related symptoms and should not be used to populate symptoms in electronic medical record–based cohorts.

## Teaser

This cohort study compares the performance of *International Classification of Diseases and Related Health Problems, Tenth Revision* (*ICD-10*) codes specific for fever, cough, and dyspnea with electronic medical record review for patients tested for coronavirus disease 2019 (COVID-19).

## Introduction

Health care organizations need rapid access to high-quality, multicenter data to support scientific discovery during the severe acute respiratory syndrome coronavirus 2 (SARS-CoV-2) pandemic, the causative agent for coronavirus disease 2019 (COVID-19). Electronic medical record (EMR) data could be repurposed to populate COVID-19 registries and surveillance systems. Several organizations are moving quickly to aggregate EMR data across multiple institutions to meet data needs.^1^ However, some critical data elements specific to COVID-19 may be unreliably captured by standard terminologies used in EMRs. The *International Statistical Classification of Diseases and Related Health Problems, Tenth Revision *(*ICD-10*) is a widely used terminology, in which each code represents a clinical concept.^2^ Some codes may lack accuracy for the intended condition,^3,4^ a challenge that is germane to COVID-19–related symptoms. The goal of this project was to compare *ICD-10* codes with manual EMR review in capturing symptoms of fever, cough, and dyspnea among patients being tested for SARS-CoV-2 infection.

## Methods

This cohort study was approved by the University of Utah institutional review board, which waived the requirement for informed consent because the study was retrospective and posed no more than minimal risk to participants. This study follows the Strengthening the Reporting of Observational Studies in Epidemiology (STROBE) reporting guideline for cohort studies.^5^

Candidate patients for this analysis included 9355 patients tested for SARS-CoV-2 at University of Utah Health from March 10 to April 6, 2020. University of Utah Health is a tertiary academic health care system in the Mountain West that includes inpatient care and regional community clinics. The system maintains an operational dashboard of all patients tested for SARS-CoV-2. These analyses were built off this dashboard, linking the medical record numbers to the Enterprise Data Warehouse (EDW) to capture *ICD-10* billing codes. The EDW aggregates data across the health system, to create a central resource for operations and research. We included anyone who was tested at our center, regardless of where the test was conducted (eg, emergency department, drive-through, or inpatient). Symptoms were not a prerequisite for testing, because institutional policy changed during the study period from only testing symptomatic patients with a known exposure to SARS-CoV-2 to testing any patient with suspected SARS-CoV-2 infection. Patients were tested using direct quantitative reverse transcriptase–polymerase chain reaction detection of SARS-CoV-2 RNA, predominantly from nasopharyngeal swabs. Serology-based testing was not used during the study period.

### Review Classification Process

The symptoms of interest were fever, cough, and dyspnea, which are common in COVID-19.^6,7,8^ A convenience sample of 2201 patient EMRs was reviewed. Early in the pandemic, a REDCap registry^9^ was prepopulated with tested patients in nearly real time, including the text from clinical notes during the 24-hour period before or after the time of the test. By default, in Python statistical software, the patients were sorted by EMR number. Each patient’s EMR was reviewed by 1 of 7 reviewers and was labeled as symptoms present, absent, or unmentioned, which served as the reference standard. After the initial review phase on March 31, 2020, we calculated the proportion of patients reviewed per day among all tested patients; additional patients were reviewed as needed to achieve approximately 20% reviewed per day (range, 18%-50%). Fewer patients were reviewed after March 31 through April 6, 2020 (171 patients), randomly selected from the registry.

### *ICD-10* Classification Process

We extracted all *ICD-10* codes associated with the SARS-CoV-2 testing visit for each patient by matching visit numbers. Codes specific for the outcomes (R50* for fever, R05* for cough, and R06.0* for dyspnea) were selected on the basis of the specifications suggested by the National COVID Cohort Collaborative.^10^ The asterisk (*) denotes that any code starting with the specified alphanumeric sequence would be included (ie, R06.03 is included for cough). Using this approach, the following codes were present in our data: R50.9, R50.81, R05, R06.02, R06.00, R06.03, and R06.09. Patients with at least 1 code in a given category were classified as having the symptom according to *ICD-10* code.

### Statistical Analysis

Sensitivity, specificity, positive predictive value (PPV), and negative predictive value (NPV) were calculated by comparing *ICD-10* codes with the reference standard; 95% CIs were calculated by bootstrapping the point estimates using random resampling and replacement to create 1000 samples of the same size as the original group. The empirical bootstrap distribution was then used to calculate the 95% CIs for each performance characteristic. Symptom present was compared with symptom absent or unmentioned, combined. Unmentioned symptoms are more likely to be absent than present, but given the uncertainty, we performed a sensitivity analysis in which unmentioned symptoms were assumed to be present.

Performance characteristics were calculated overall and stratified by subgroups, including SARS-CoV-2 test result, sex, age group (<50, 50-64, and >64 years), and inpatient status. SARS-CoV-2 test results and demographic characteristics were captured through routine documentation for clinical care and were extracted from documented values in the EDW. Patients could be classified as inpatient status in 1 of 2 ways: the test was performed in an inpatient unit or the patient was hospitalized within 14 days of the testing period. We chose this approach because testing frequently occurs in drive-through units, with distinct visit numbers. However, if a patient is ill enough, they may be hospitalized soon after under a different visit number. Clinically, these patients should be classified as inpatients, as a marker of disease severity, which was the rationale for our approach.

We compared the observed number of false-positive, false-negative, true-positive, and true-negative *ICD-10–*based classifications for each subgroup using 1-sided Pearson χ^2^ tests. A *P* < .05 was considered significant, and all analyses were performed using Python statistical software version 3.6 (Python). Data analysis was performed in April 2020.

## Results

Among 2201 patients tested for SARS-CoV-2 whose EMRs were reviewed for this study, the mean (SD) age was 42 (17) years (median age, 40 years; interquartile range, 29-54 years); 1201 (55%) were female, 1569 (71%) were White, and 282 (13%) were Hispanic or Latino. Most patients (2007 patients [91%]), were tested in an outpatient setting, whereas 156 (7%) were tested in the emergency department and 65 (3%) were tested in an inpatient setting. The median number of *ICD-10* codes associated with each testing visit was 1 (interquartile range, 1-3 codes). Performance characteristics were poor for all symptoms (Table 1). On the basis of EMR review, the reference standard, fever was present in 1444 patients (66%), cough was present in 1930 patients (88%), and dyspnea was present in 1399 patients (64%). Fever was unmentioned in 6.5%, cough in 3.9%, and dyspnea in 10.3% of patient EMRs. *ICD-10* codes had poor sensitivity and NPV for all of the symptoms compared with the reference standard. The sensitivity of *ICD-10* codes was 0.26 (95% CI, 0.24-0.29) for fever, 0.44 (95% CI, 0.42-0.46) for cough, and 0.24 (95% CI, 0.22-0.26) for dyspnea. NPV was poor for all symptoms: 0.41 (95% CI, 0.39-0.43) for fever, 0.18 (95% CI, 0.16-0.20) for cough, and 0.42 (95% CI, 0.40-0.44) for dyspnea. Specificity was 0.98 (95% CI, 0.96-0.99) for fever, 0.88 (95% CI, 0.84-0.92) for cough, and 0.97 (95% CI, 0.96-0.98) for dyspnea. PPV was 0.96 (95% CI, 0.93-0.97) for fever, 0.96 (95% CI, 0.95-0.97) for cough, and 0.93 (95% CI, 0.90-0.96) for dyspnea.

**Table 1.** Performance Characteristics of *International Statistical Classification of Diseases and Related Health Problems, Tenth Revision* Codes for Identifying Fever, Cough, and Dyspnea Among Patients Tested for Coronavirus Disease 2019^a^

Symptom^b^	Point estimate (95% CI)			
	Sensitivity	Specificity	PPV	NPV
Fever	0.26 (0.24-0.29)	0.98 (0.96-0.99)	0.96 (0.93-0.97)	0.41 (0.39-0.43)
Cough	0.44 (0.42-0.46)	0.88 (0.84-0.92)	0.96 (0.95-0.97)	0.18 (0.16-0.20)
Dyspnea	0.24 (0.22-0.26)	0.97 (0.96-0.98)	0.93 (0.90-0.96)	0.42 (0.40-0.44)

Abbreviations: NPV, negative predictive value; PPV, positive predictive value.

^a^ The total number of patients was 2201.

^b^ *International Statistical Classification of Diseases and Related Health Problems, Tenth Revision* codes were R50* for fever, R05* for cough, and R06.0* for dyspnea.

In our sensitivity analysis, we assumed that all unmentioned symptoms were present. The recalculated *ICD-10* performance characteristics were a sensitivity of 0.25 (95% CI, 0.22-0.27) for fever, 0.43 (95% CI, 0.41-0.45) for cough, and 0.21 (95% CI, 0.19-0.23) for dyspnea. NPV was poor for all symptoms, with 0.33 (95% CI, 0.32-0.36) for fever, 0.12 (95% CI, 0.11-0.14) for cough, and 0.30 (95% CI, 0.28-0.32) for dyspnea. Specificity and PPV ranged from 0.89 to 0.99 for all symptoms (95% CI, 0.84-0.99).

Table 2, Table 3, and Table 4 display the performance characteristics for each symptom in the prespecified subgroups. Sensitivity was low for all symptoms in all subgroups (range, 0.17-0.45). The observed values of false-positives, false-negatives, true-positives, and true-negatives differed between inpatients and outpatients for fever (χ^2^ = 41.30; *P* < .001) and dyspnea (χ^2^ = 14.25; *P* = .003) but not cough (χ^2^ = 5.13; *P* = .16). Statistically significant differences in the same observed values were found between age subgroups for fever (χ^2^ = 42.63; *P* < .001) and dyspnea (χ^2^ = 14.77; *P* = .02) but not cough (χ^2^ = 6.28; *P* = .39). Here, we report the χ^2^ statistic and *P* value; see eTable 1, eTable 2, and eTable 3 in the Supplement for the contingency tables used to generate these results. High false-negative rates were the main contributor to poor *ICD-10* code performance. The proportion of patients with a false-negative *ICD-10* code result ranged from 35.8% for fever among patients older than 64 years to 54.5% for fever among patients who tested positive for SARS-CoV-2 infection.

**Table 2.** Performance Characteristics of *International Statistical Classification of Diseases and Related Health Problems, Tenth Revision* Codes for Identifying Fever Among Patient Subgroups Tested for Coronavirus Disease 2019

Subgroup	Patients, No. (N = 2201)	Point estimate (95% CI)			
		Sensitivity	Specificity	PPV	NPV
SARS-CoV-2 test result					
Positive	156	0.26 (0.17-0.35)	0.95 (0.88-1.00)	0.94 (0.85-1.00)	0.31 (0.23-0.40)
Negative	2045	0.26 (0.24-0.29)	0.98 (0.97-0.99)	0.96 (0.94-0.98)	0.42 (0.39-0.44)
Sex					
Male	1000	0.27 (0.24-0.31)	0.97 (0.95-0.99)	0.95 (0.91-0.97)	0.43 (0.40-0.47)
Female	1201	0.26 (0.23-0.29)	0.98 (0.97-0.99)	0.96 (0.94-0.99)	0.39 (0.36-0.42)
Age group, y^a^					
<50	1494	0.27 (0.24-0.29)	0.98 (0.97-0.99)	0.97 (0.95-0.99)	0.37 (0.34-0.40)
50-64	436	0.24 (0.18-0.29)	0.98 (0.95-0.99)	0.94 (0.88-0.99)	0.45 (0.40-0.50)
>64	271	0.30 (0.22-0.38)	0.96 (0.93-0.99)	0.89 (0.80-0.98)	0.57 (0.50-0.63)
Clinical setting^a^					
Inpatient	97	0.39 (0.28-0.52)	0.83 (0.71-0.95)	0.80 (0.67-0.93)	0.45 (0.33-0.58)
Outpatient	2104	0.26 (0.24-0.28)	0.98 (0.97-0.99)	0.97 (0.95-0.98)	0.41 (0.39-0.43)

Abbreviations: NPV, negative predictive value; PPV, positive predictive value; SARS-CoV-2, severe acute respiratory syndrome coronavirus 2.

^a^ Indicates* International Statistical Classification of Diseases and Related Health Problems, Tenth Revision* codes for fever performed differently for the given symptom in the specified subgroup. Comparison was based on a χ^2^ test of observed vs expected number of false-positive, false-negative, true-positive, and true-negative code-based classifications. *P* < .05 was considered significant.

**Table 3.** Performance Characteristics of *International Statistical Classification of Diseases and Related Health Problems, Tenth Revision* Codes for Identifying Cough Among Patient Subgroups Tested for Coronavirus Disease 2019

Subgroup^a^	Patients, No. (N = 2201)	Point estimate (95% CI)			
		Sensitivity	Specificity	PPV	NPV
SARS-CoV-2 test result					
Positive	156	0.44 (0.36-0.53)	0.79 (0.62-0.94)	0.21 (0.85-0.98)	0.20 (0.13-0.29)
Negative	2045	0.44 (0.42-0.47)	0.89 (0.85-0.92)	0.97 (0.95-0.98)	0.18 (0.16-0.20)
Sex					
Male	1000	0.43 (0.40-0.46)	0.88 (0.82-0.93)	0.96 (0.93-0.98)	0.20 (0.17-0.23)
Female	1201	0.45 (0.42-0.48)	0.88 (0.82-0.93)	0.97 (0.95-0.98)	0.17 (0.14-0.19)
Age group, y					
<50	1494	0.45 (0.42-0.48)	0.88 (0.84-0.93	0.96 (0.95-0.98)	0.19 (0.16-0.22)
50-64	436	0.43 (0.39-0.49)	0.81 (0.7-0.91)	0.94 (0.91-0.98)	0.16 (0.12-0.21)
>64	271	0.41 (0.35-0.47)	0.97 (0.88-1.0)	0.99 (0.97-1.0)	0.17 (0.11-0.22)
Clinical setting					
Inpatient	97	0.41 (0.30-0.51)	1.00 (1.00-1.00)	1.00 (1.00-1.00)	0.25 (0.15-0.36)
Outpatient	2104	0.44 (0.42-0.47)	0.87 (0.83-0.91)	0.96 (0.95-0.97)	0.18 (0.16-0.2)

Abbreviation: SARS-CoV-2, severe acute respiratory syndrome coronavirus 2.

^a^ *International Statistical Classification of Diseases and Related Health Problems, Tenth Revision* codes for cough performed similarly for all subgroups. Comparison was based on a χ^2^ test of observed vs expected number of false positive, false negative, true positive, and true negative ICD-10 based classifications; *P* < .05 was considered significant.

**Table 4.** Performance Characteristics of *International Statistical Classification of Diseases and Related Health Problems, Tenth Revision *Codes for Identifying Dyspnea Among Patient Subgroups Tested for Coronavirus Disease 2019

Subgroup	Patients, No. (N = 2201)	Point estimate (95% CI)			
		Sensitivity	Specificity	PPV	NPV
SARS-CoV-2 test result^a^					
Positive	156	0.17 (0.09-0.25)	0.97 (0.93-1.00)	0.87 (0.67-1.00)	0.54 (0.46-0.61)
Negative	2045	0.24 (0.22-0.27)	0.97 (0.96-0.98)	0.94 (0.91-0.96)	0.41 (0.39-0.44)
Sex^a^					
Male	1000	0.25 (0.21-0.28)	0.98 (0.97-0.99)	0.96 (0.92-0.99)	0.45 (0.42-0.49)
Female	1201	0.23 (0.20-0.26)	0.96 (0.94-0.98)	0.91 (0.87-0.95)	0.40 (0.36-0.43)
Age group, y^a^					
<50	1494	0.22 (0.19-0.25)	0.98 (0.96-0.99)	0.94 (0.91-0.97)	0.40 (0.38-0.43)
50-64	436	0.27 (0.21-0.32)	0.98 (0.95-0.99)	0.95 (0.89-0.99)	0.45 (0.40-0.50)
>64	271	0.29 (0.22-0.36)	0.94 (0.89-0.98)	0.87 (0.76-0.95)	0.48 (0.42-0.55)
Clinical setting^a^					
Inpatient	97	0.36 (0.25-0.48)	0.89 (0.76-1.0)	0.89 (0.77-1.0)	0.36 (0.25-0.49)
Outpatient	2104	0.23 (0.21-0.25)	0.97 (0.96-0.98)	0.94 (0.91-0.96)	0.42 (0.40-0.45)

Abbreviations: NPV, negative predictive value; PPV, positive predictive value; SARS-CoV-2, severe acute respiratory syndrome coronavirus 2.

^a^ *International Statistical Classification of Diseases and Related Health Problems, Tenth Revision* codes for dyspnea performed differently for the given symptom in the specified subgroup. Comparison was based on a χ^2^ test of observed vs expected number of false-positive, false-negative, true-positive, and true-negative code-based classifications. *P* < .05 was considered significant.

## Discussion

Symptoms are an essential part of data collection for SARS-CoV-2 and COVID-19 surveillance and research, but symptom-specific *ICD-10* codes lack sensitivity and fail to capture many patients with relevant symptoms; the false-negative rate is unacceptably high. Common data models and other aggregation tools rely heavily on *ICD-10* codes to capture clinical concepts; inaccuracy has implications for any downstream scientific discovery or surveillance.^10,11^ For example, symptom surveillance could be important to detect subsequent waves of COVID-19, similar to the US Outpatient Influenza-Like Illness Surveillance Network.^12^ A substantial number of patients would be missed if *ICD-10* codes were used for this task.

*ICD-10* codes are known to lack accuracy for clinical diagnoses and concepts. For example, *ICD-10* codes perform poorly to identify patients with atrial fibrillation, with a sensitivity of 88% and a specificity of 42%.^4^ Similar inaccuracies have been reported for other conditions, such as stroke and acute kidney injury.^13,14^ Our work represents clinician documentation of symptoms, and clinicians may not document all symptoms for all patients, particularly when patient volume is high or in drive-through testing scenarios. In other words, clinician documentation is not necessarily the “gold standard,” but rather a reference standard. Other strategies include checklist type data entry to support standardized data collection or capturing symptoms directly from the patient. Several public health agencies are developing smartphone applications that allow people to report symptoms directly to appropriate officials.^15^ For health care systems, patient-reported outcomes may allow more reliable symptom capture, without reliance on billing codes or clinician documentation.^16^

Our findings highlight the importance of quality control in COVID-19 data aggregation, which has become increasingly important with recent high-profile journal retractions.^17^ Critical data elements require careful validation to ensure that discoveries translate into effective interventions that reduce morbidity and mortality. As with many aspects of this pandemic, we must pay careful attention to socioeconomically vulnerable populations, including racial minorities, rural patients, and low-income patients, for whom the gap between *ICD-10* coding and clinical reality could be greater.^18,19^

### Limitations

This study has limitations that should be considered. Our study included only a single center; other centers may have different *ICD-10* performance characteristics. Our study also uses data from early in the pandemic, and performance characteristics could change over time. Furthermore, as noted earlier, clinicians may not document all symptoms in every case. Although we did not adjust for multiple comparisons, *ICD-10* code performance is so poor that adjustment is unlikely to alter the interpretation of these results. Each case was reviewed by a single individual; because of the low complexity of the studied concepts (presence or absence of fever, cough, and dyspnea), a single-reviewer system is likely sufficient in this context. In addition, the reviewed cases were not selected randomly but rather in nearly real time as the pandemic situation evolved. This approach could introduce a bias but, again, given how poorly the codes perform, we doubt that a randomly selected sample would alter the results. Still, future studies should prespecify a plan for data validation, with a focus on sampling racial and ethnic minorities to ensure generalizable results.

## Conclusions

Rapid access to well-characterized, large SARS-CoV-2 and COVID-19 cohorts is critical for scientific discovery. *ICD-10* codes are a standard terminology and are attractive for data aggregation because they are uniformly used among health care systems. However, these codes perform poorly in capturing COVID-19–related symptoms. Our findings highlight the critical need for meticulous data validation to feed multicenter registries built from EMRs. Reliable, accurate data are the foundation of scientific discovery; the right data lead to the right solutions.

## Notes

Supplement. **eTable 1. **Contingency Table of *ICD-10* Codes for Identifying Fever Among Patient Subgroups Tested for COVID-19 **eTable 2. **Contingency Table of *ICD-10* Codes for Identifying Cough Among Patient Subgroups Tested for COVID-19 **eTable 3. **Contingency Table of *ICD-10* Codes for Identifying Dyspnea Among Patient Subgroups Tested for COVID-19
