# Supplementary material for: Comparison of International Classification of Diseases and Related Health Problems, Tenth Revision Codes With Electronic Medical Records Among Patients With Symptoms of Coronavirus Disease 2019
Source: JAMA Netw Open. 2020 Aug 14;3(8):e2017703. doi: 10.1001/jamanetworkopen.2020.17703 (PMC7428802; doi:10.1001/jamanetworkopen.2020.17703)
Supplement: Supplement. — eTable 1. Contingency Table of ICD-10 Codes for Identifying Fever Among Patient Subgroups Tested for COVID-19 eTable 2. Contingency Table of ICD-10 Codes for Identifying Cough Among Patient Subgroups Tested for COVID-19 eTable 3. Contingency Table of ICD-10 Codes for Identifying Dyspnea Among Patient Subgroups Tested for COVID-19 [file jamanetwopen-3-e2017703-s001.pdf]

Supplementary Online Content

Crabb BT, Lyons A, Bale M, et al. Comparison of *International Classification of Diseases and Related Health Problems, Tenth Revision* codes with electronic medical records among patients with symptoms of coronavirus disease 2019. *JAMA Netw Open*. 2020;3(8):e2017703. doi:10.1001/jamanetworkopen.2020.17703

eTable 1. Contingency Table of *ICD-10* Codes for Identifying Fever Among Patient Subgroups Tested for COVID-19
eTable 2. Contingency Table of *ICD-10* Codes for Identifying Cough Among Patient Subgroups Tested for COVID-19
eTable 3. Contingency Table of *ICD-10* Codes for Identifying Dyspnea Among Patient Subgroups Tested for COVID-19

This supplementary material has been provided by the authors to give readers additional information about their work.

© 2020 Crabb BT et al. *JAMA Network Open.*

eTable 1. Contingency Table of *ICD-10* Codes for Identifying Fever Among Patient Subgroups Tested for COVID-19

	**Classification Outcome**			
**Subgroup**	**True Positives**	**False Positives**	**False Negatives**	**True Negatives**
**COVID-19 Positive**				
Positive (n=156)	30	2	85	39
Negative (n=2045)	352	16	977	700
**Sex**				
Male (n=1000)	173	10	462	355
Female (n=1201)	209	8	600	384
**Age Group***				
<50 (n=1494)	277	9	762	446
50-64 (n=436)	63	4	203	166
>64 (n=271)	42	5	97	127
**Clinical Setting***				
Inpatient (n=97)	24	6	37	30
Outpatient (n=2104)	358	12	1025	709

**Indicates ICD-10 codes performed differently for the given symptom in the specified subgroup. Comparison was based on a chi-squared test of observed versus expected number of false positive, false negative, true positive, and true negative ICD-10 based classifications; p<0.05 was considered significant.*
ICD-10=International Classification of Diseases Version 10

© 2020 Crabb BT et al. *JAMA Network Open.*

eTable 2. Contingency Table of *ICD-10* Codes for Identifying Cough Among Patient Subgroups Tested for COVID-19

	Classification Outcome			
**Subgroup**	**True Positives**	**False Positives**	**False Negatives**	**True Negatives**
**COVID-19 Positive**				
Positive (n=156)	58	5	74	19
Negative (n=2045)	794	28	1004	219
**Sex**				
Male (n=1000)	372	17	491	120
Female (n=1201)	480	16	587	118
**Age Group**				
<50 (n=1494)	586	22	719	167
50-64 (n=436)	167	10	217	42
>64 (n=271)	99	1	142	29
**Clinical Setting**				
Inpatient (n=97)	33	0	48	16
Outpatient (n=2104)	819	33	1030	222

**Indicates ICD-10 codes performed differently for the given symptom in the specified subgroup. Comparison was based on a chi-squared test of observed versus expected number of false positive, false negative, true positive, and true negative ICD-10 based classifications; p<0.05 was considered significant.*

ICD-10=International Classification of Diseases Version 10

© 2020 Crabb BT et al. *JAMA Network Open.*

eTable 3. Contingency Table of *ICD-10* Codes for Identifying Dyspnea Among Patient Subgroups Tested for COVID-19

	**Classification Outcome**			
**Subgroup**	**True Positives**	**False Positives**	**False Negatives**	**True Negatives**
**COVID-19 Positive***				
Positive (n=156)	13	2	65	76
Negative (n=2045)	319	22	1002	702
**Sex***				
Male (n=1000)	152	7	462	379
Female (n=1201)	180	17	605	399
**Age Group***				
<50 (n=1494)	214	13	756	511
50-64 (n=436)	72	4	198	162
>64 (n=271)	46	7	113	105
**Clinical Setting***				
Inpatient (n=97)	25	3	44	25
Outpatient (n=2104)	307	21	1023	753

**Indicates ICD-10 codes performed differently for the given symptom in the specified subgroup. Comparison was based on a chi-squared test of observed versus expected number of false positive, false negative, true positive, and true negative ICD-10 based classifications; p<0.05 was considered significant.*

ICD-10=International Classification of Diseases Version 10

© 2020 Crabb BT et al. *JAMA Network Open.*
